# Supplementary figures and images for: Metabolic profile alterations in juvenile rats with bladder overactivity induced by short-term high-fructose intake
Source: PeerJ. 2025 Oct 13;13:e20186. doi: 10.7717/peerj.20186 (PMC12530204; doi:10.7717/peerj.20186)

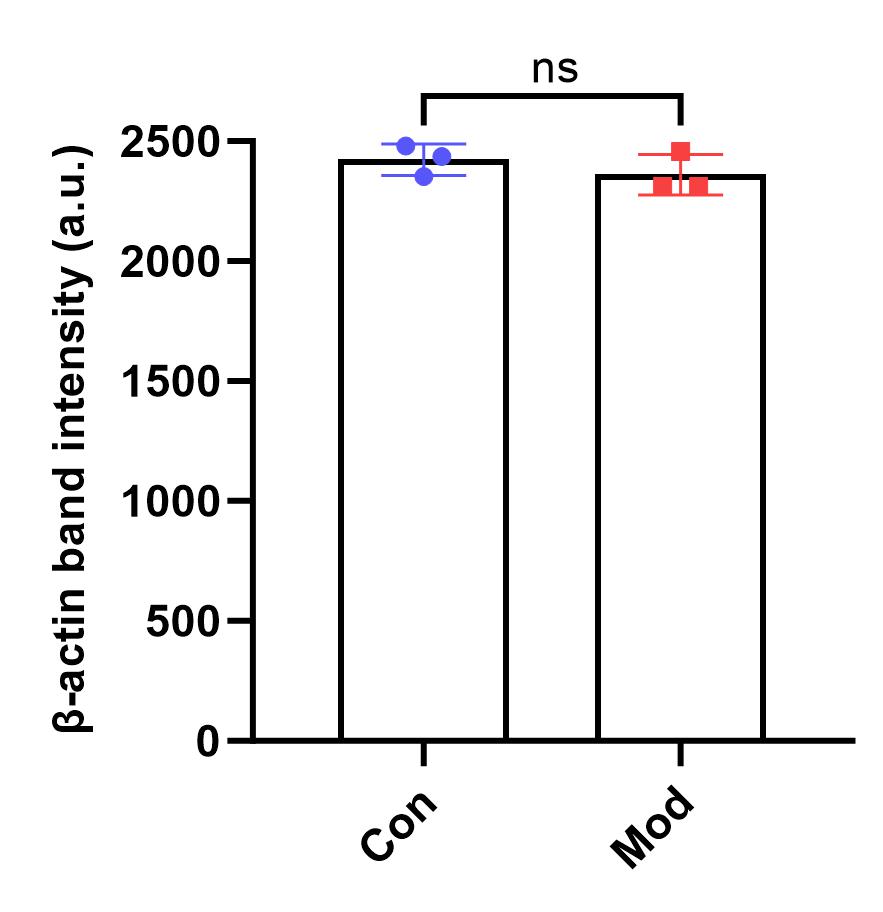

Supplement: Supplemental Information 1 [file peerj-13-20186-s001.jpg]

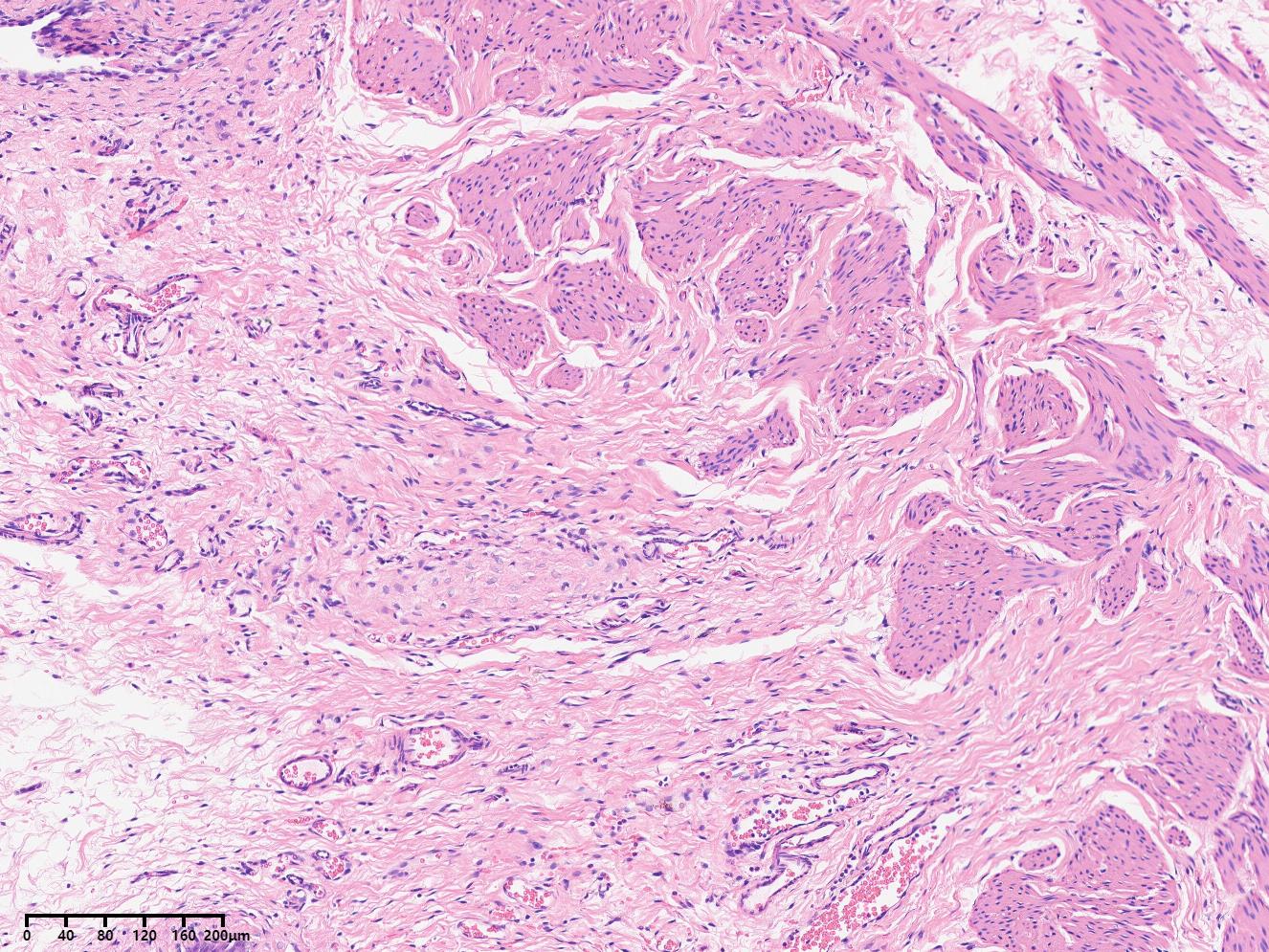

Supplement: Supplemental Information 4 [file peerj-13-20186-s004.zip › Raw date-HE and Immunohistochemical staining for Figure 5/HE staining/HE-Con/Con-1-100X.jpg]

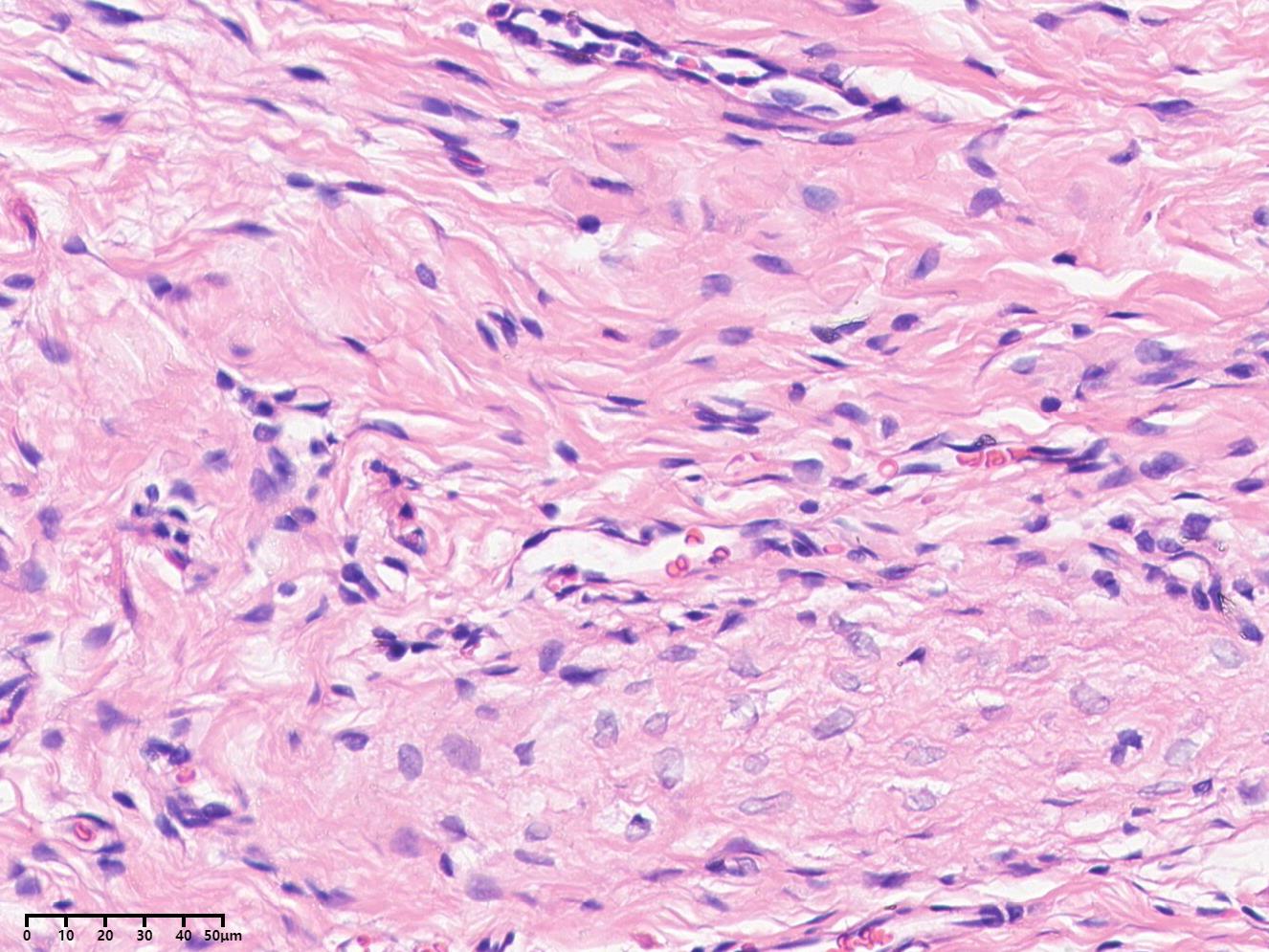

Supplement: Supplemental Information 4 [file peerj-13-20186-s004.zip › Raw date-HE and Immunohistochemical staining for Figure 5/HE staining/HE-Con/Con-1-400X.jpg]

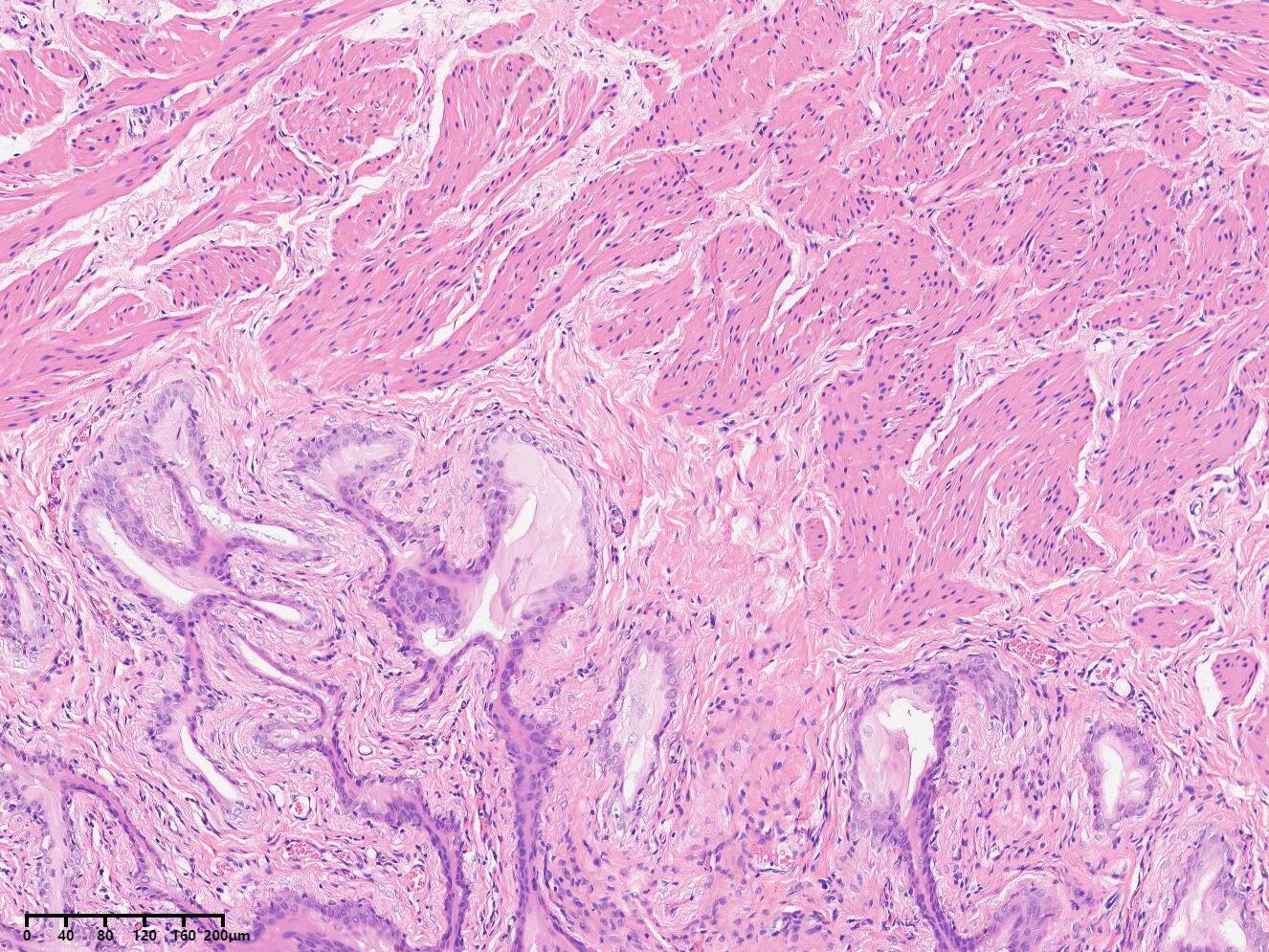

Supplement: Supplemental Information 4 [file peerj-13-20186-s004.zip › Raw date-HE and Immunohistochemical staining for Figure 5/HE staining/HE-Mod/Mod-1-100X.jpg]

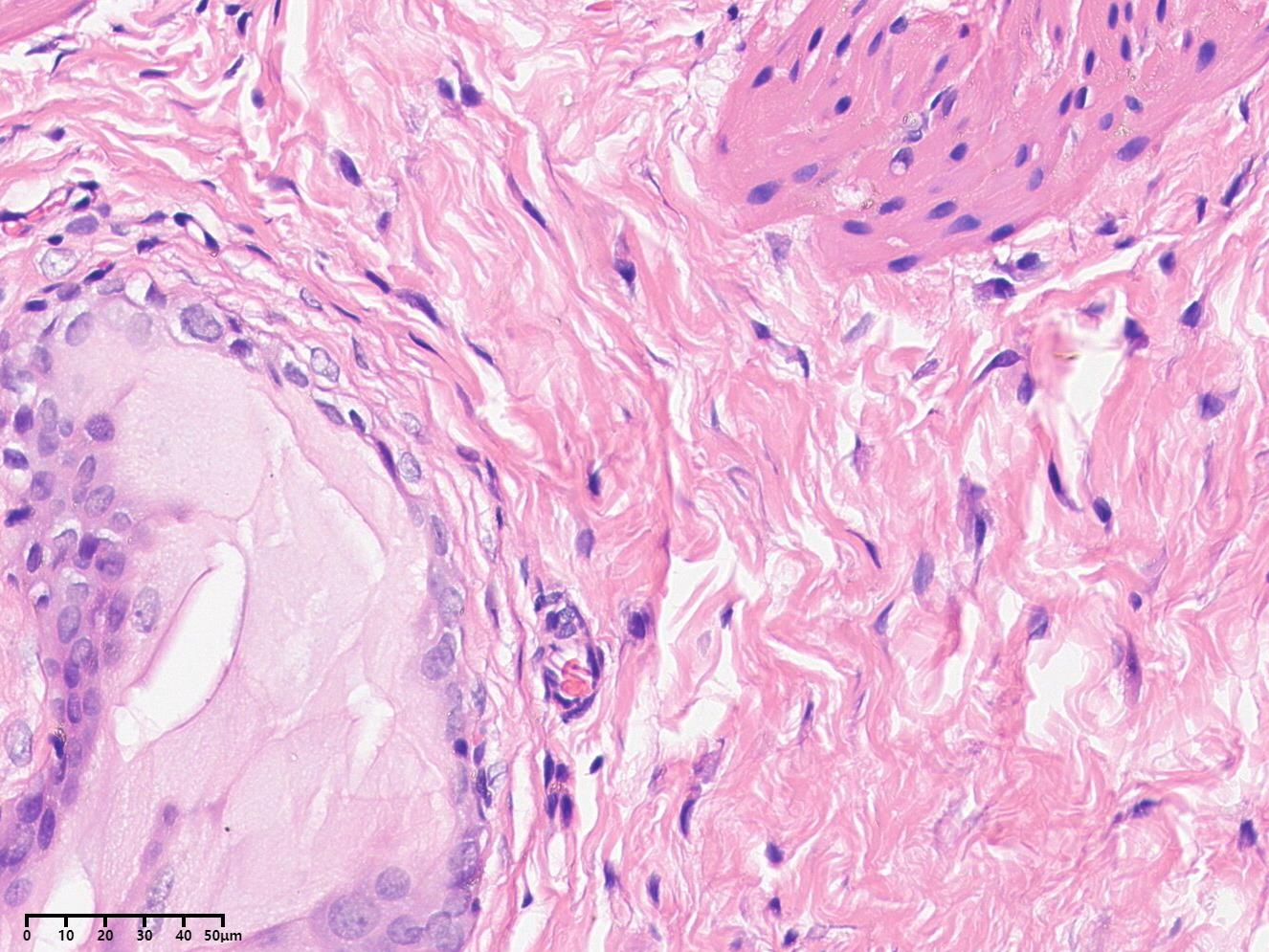

Supplement: Supplemental Information 4 [file peerj-13-20186-s004.zip › Raw date-HE and Immunohistochemical staining for Figure 5/HE staining/HE-Mod/Mod-1-400X.jpg]

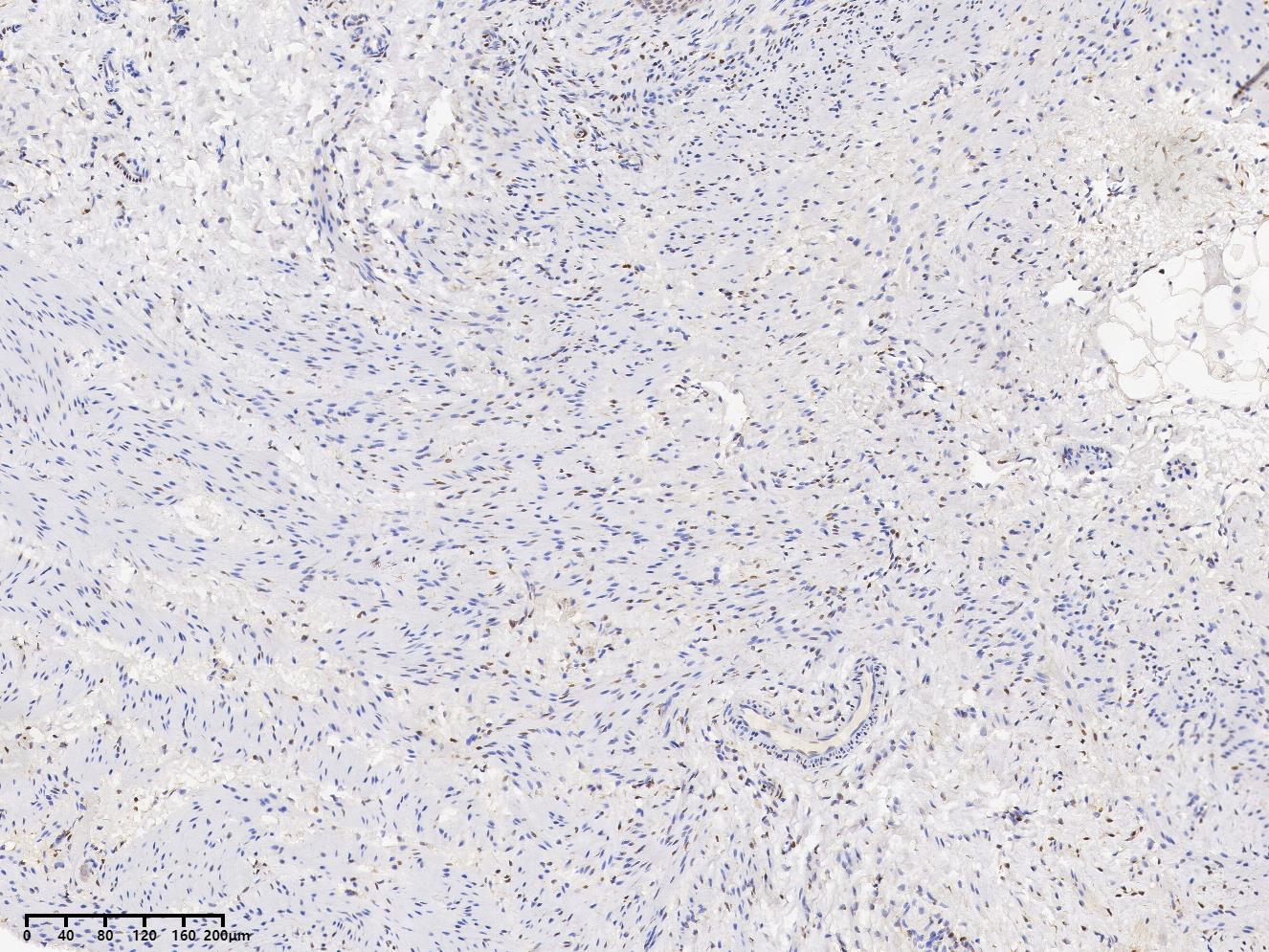

Supplement: Supplemental Information 4 [file peerj-13-20186-s004.zip › Raw date-HE and Immunohistochemical staining for Figure 5/Immunohistochemical staining/MYH-10/Con-1/Con-1-100X.jpg]

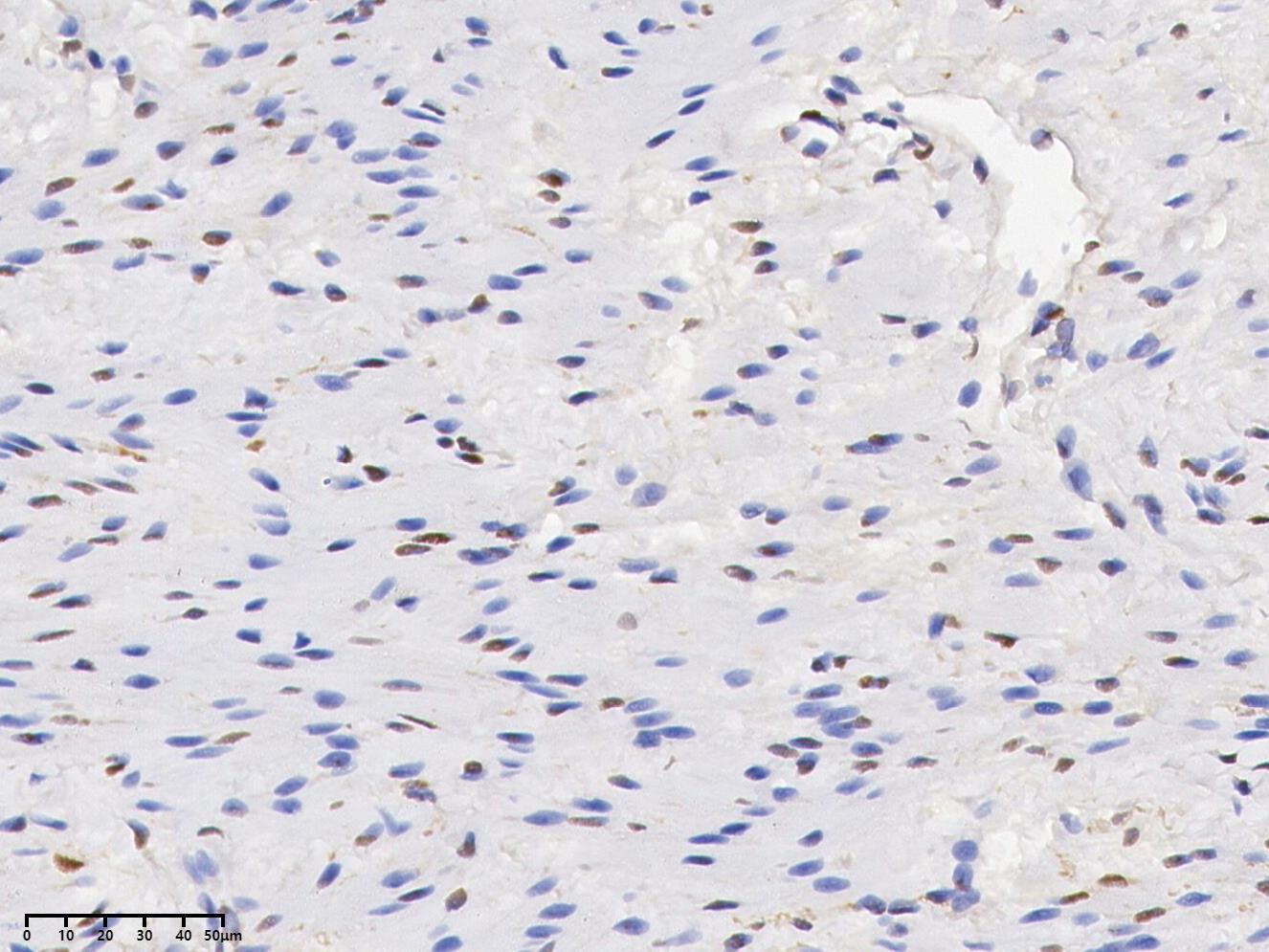

Supplement: Supplemental Information 4 [file peerj-13-20186-s004.zip › Raw date-HE and Immunohistochemical staining for Figure 5/Immunohistochemical staining/MYH-10/Con-1/Con-1-400X.jpg]

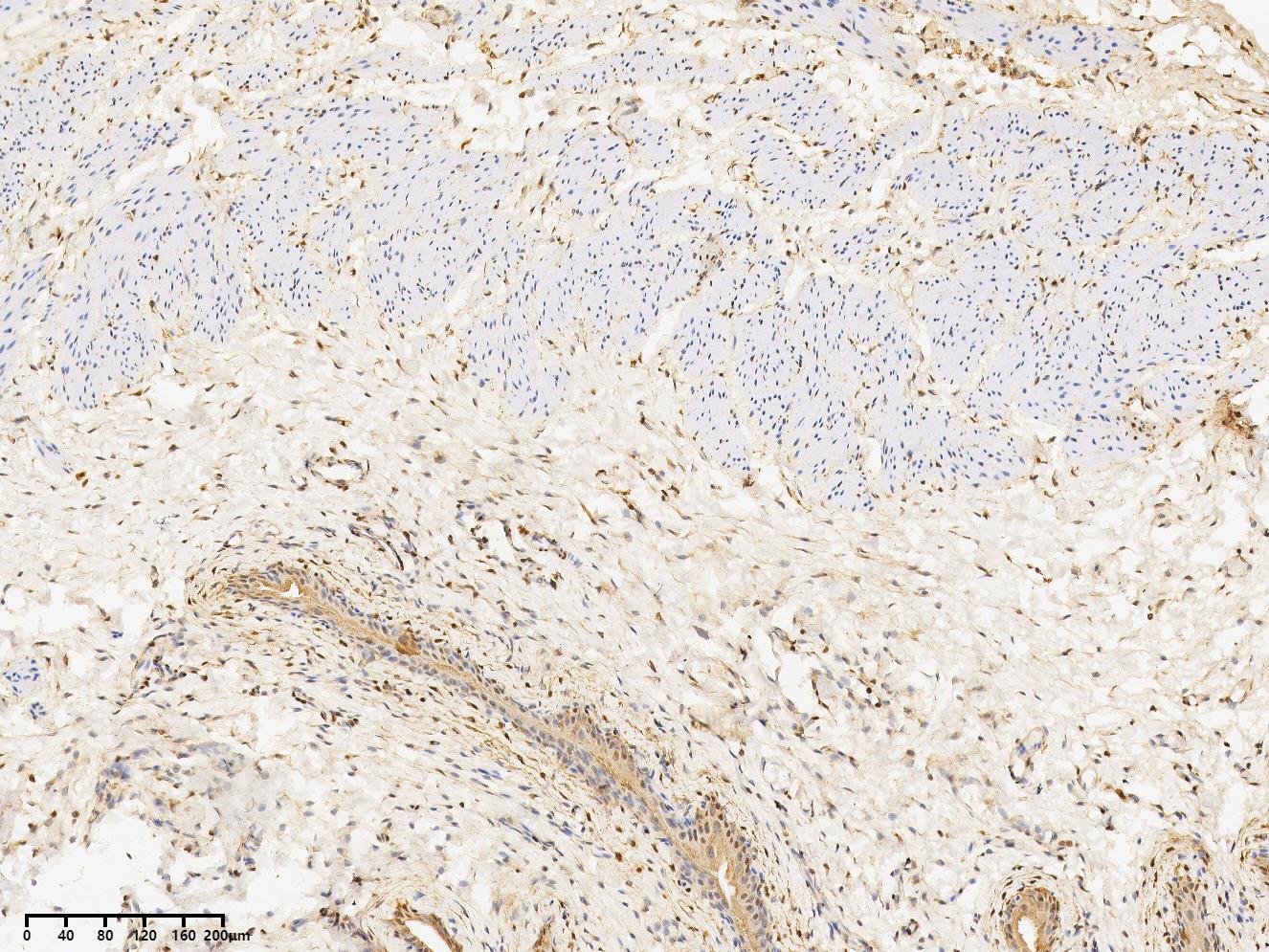

Supplement: Supplemental Information 4 [file peerj-13-20186-s004.zip › Raw date-HE and Immunohistochemical staining for Figure 5/Immunohistochemical staining/MYH-10/Mod/Mod-1-100X.jpg]

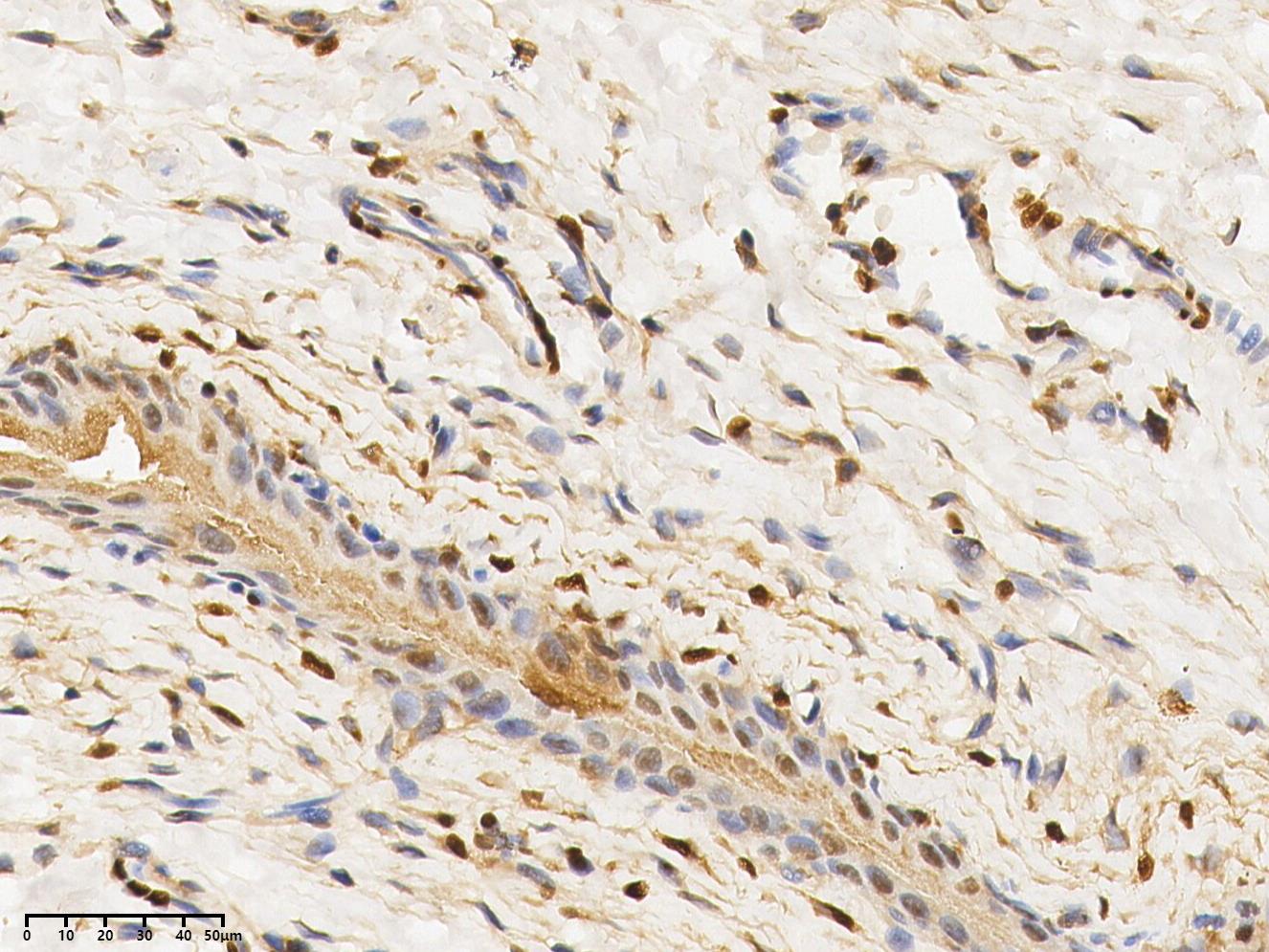

Supplement: Supplemental Information 4 [file peerj-13-20186-s004.zip › Raw date-HE and Immunohistochemical staining for Figure 5/Immunohistochemical staining/MYH-10/Mod/Mod-1-400X.jpg]

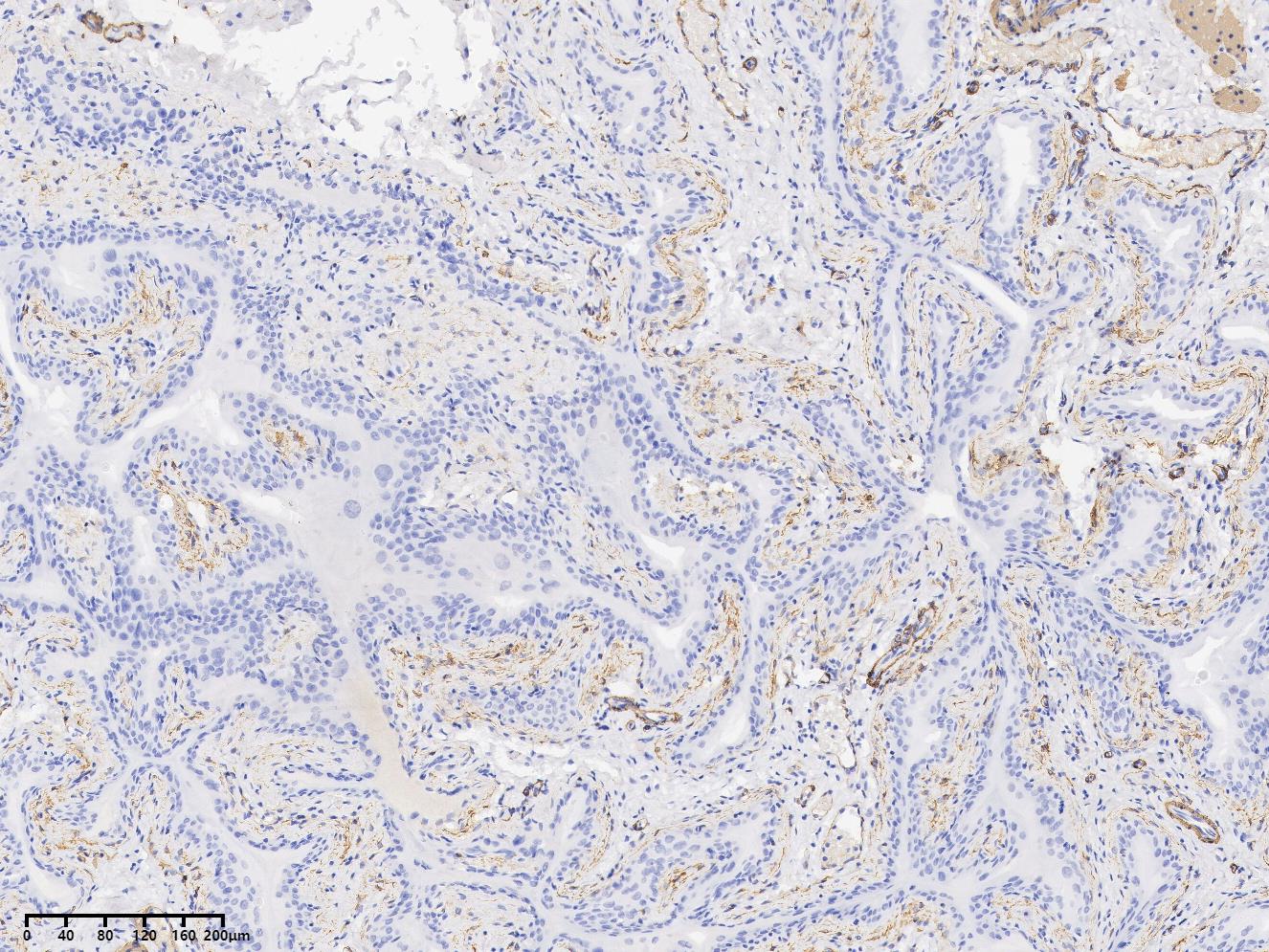

Supplement: Supplemental Information 4 [file peerj-13-20186-s004.zip › Raw date-HE and Immunohistochemical staining for Figure 5/Immunohistochemical staining/SMA/Con-1/Con-1-100X.jpg]

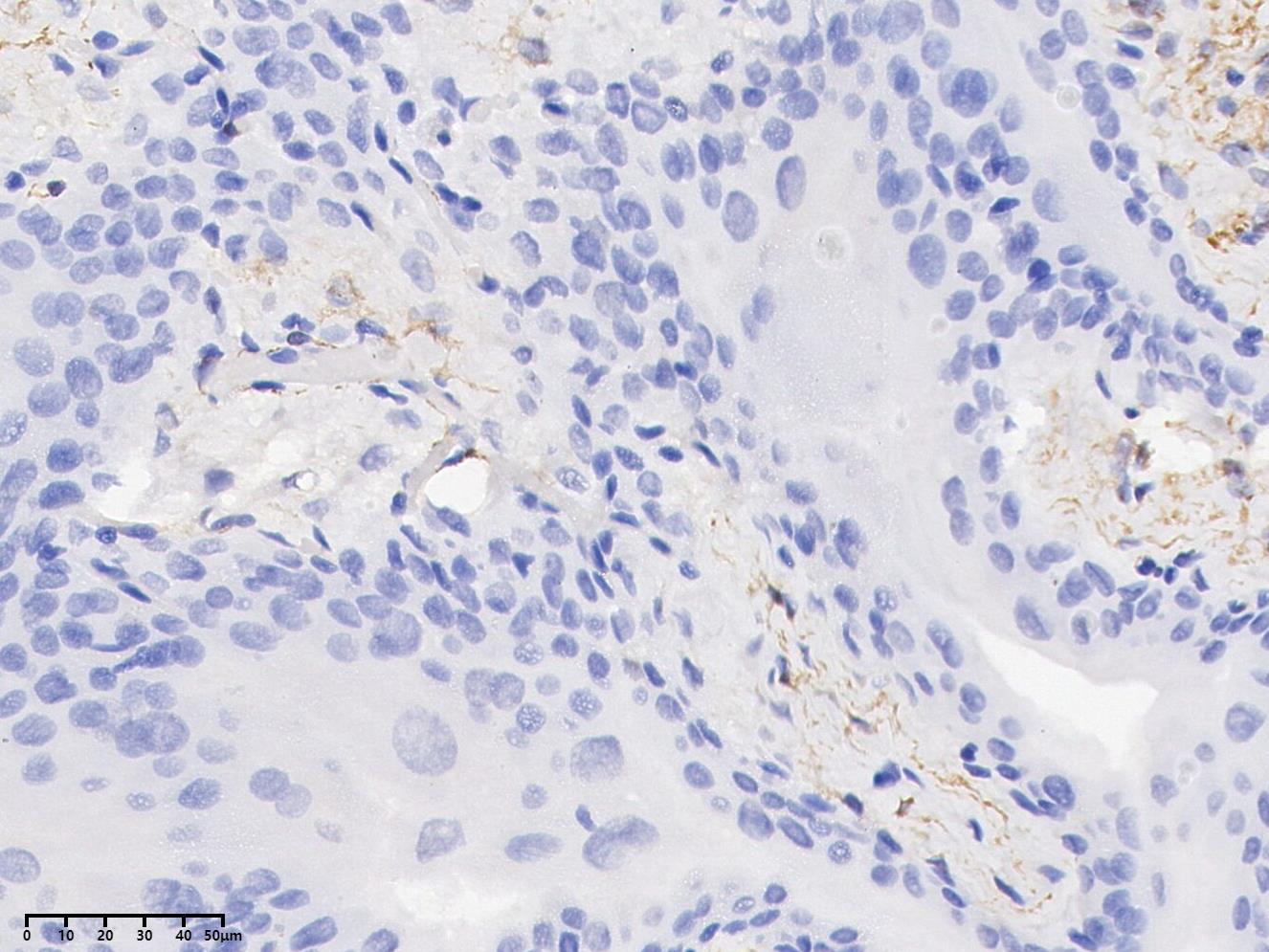

Supplement: Supplemental Information 4 [file peerj-13-20186-s004.zip › Raw date-HE and Immunohistochemical staining for Figure 5/Immunohistochemical staining/SMA/Con-1/Con-1-400X.jpg]

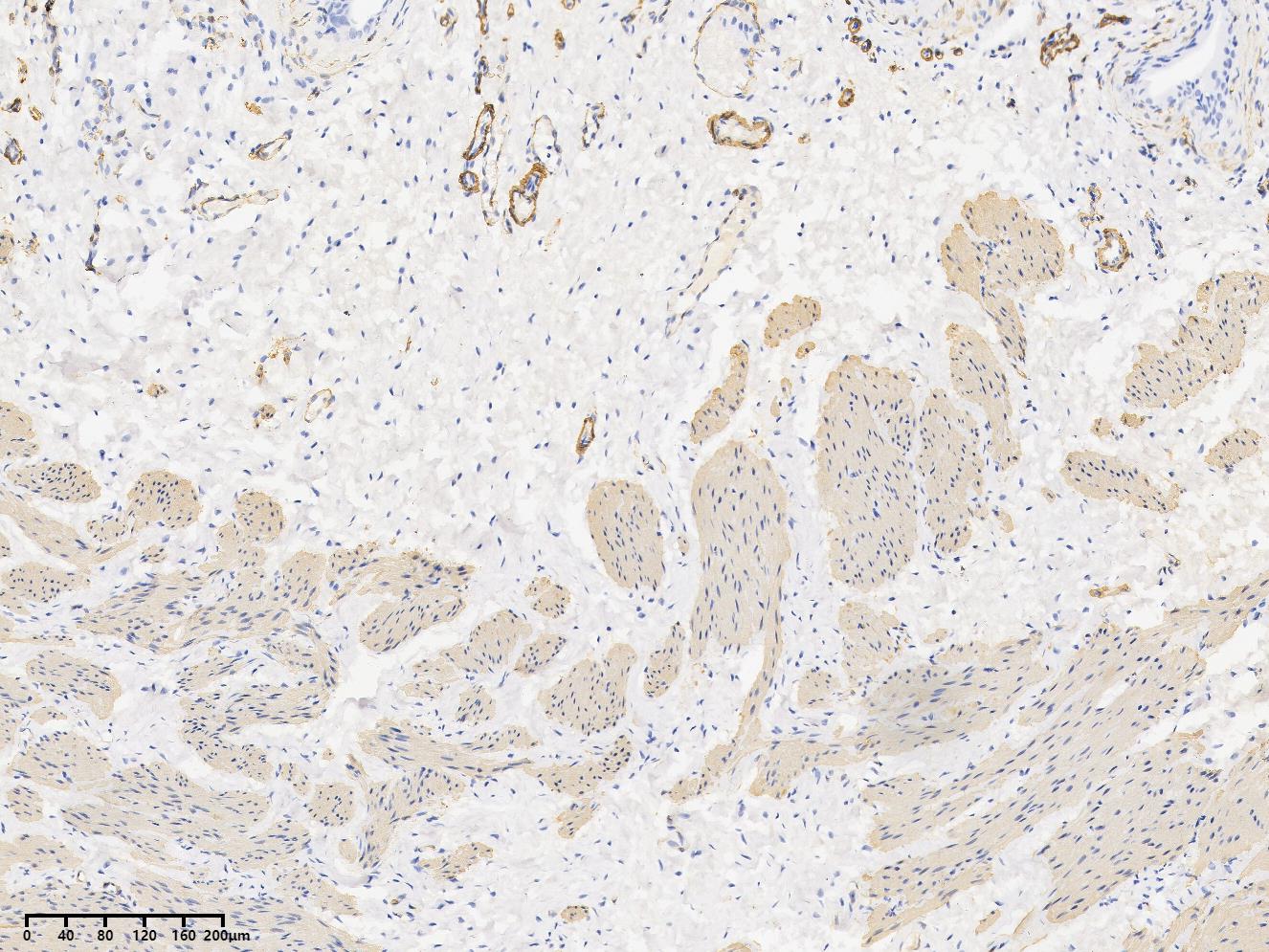

Supplement: Supplemental Information 4 [file peerj-13-20186-s004.zip › Raw date-HE and Immunohistochemical staining for Figure 5/Immunohistochemical staining/SMA/Mod/Mod-1-100X.jpg]

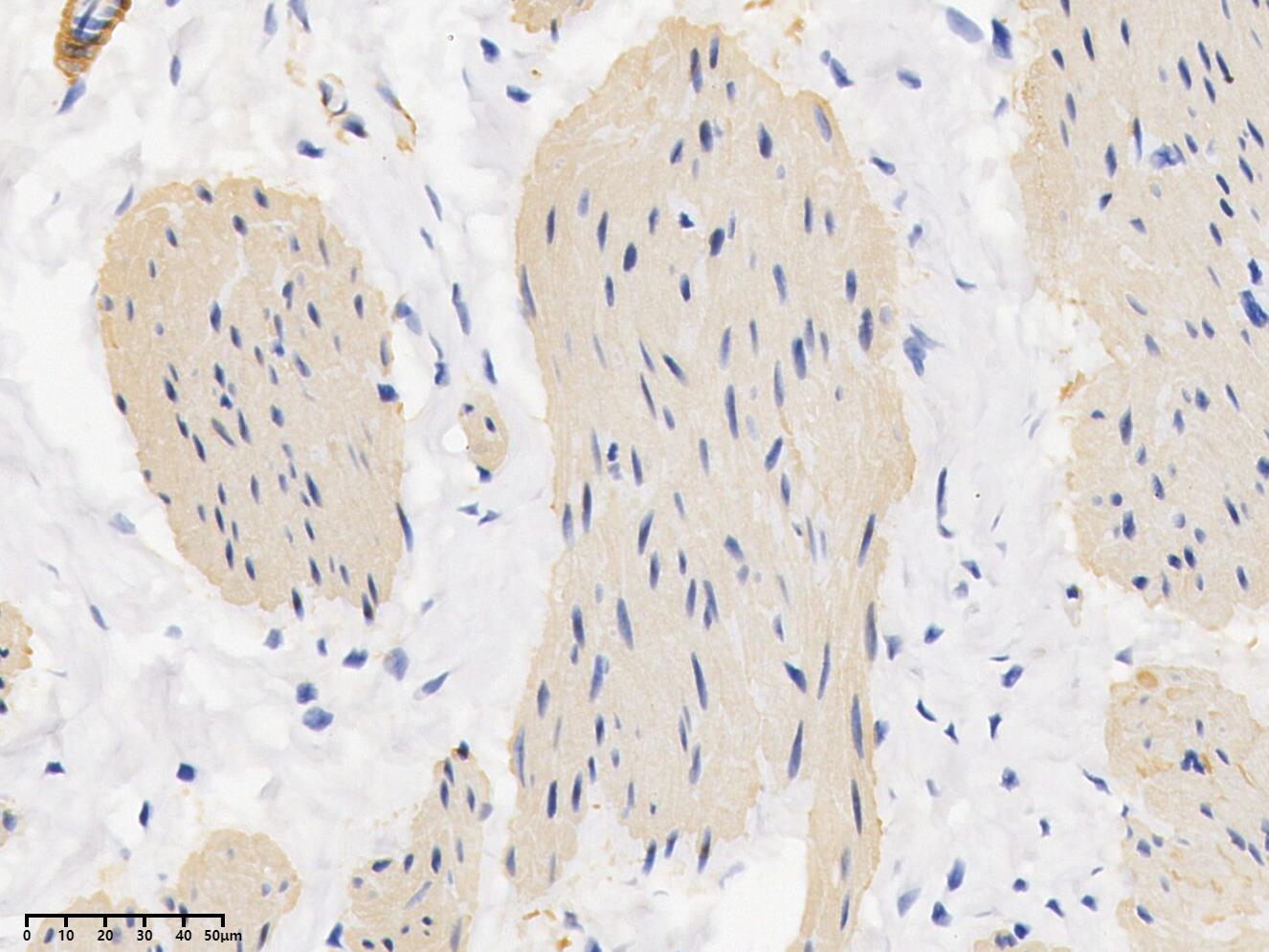

Supplement: Supplemental Information 4 [file peerj-13-20186-s004.zip › Raw date-HE and Immunohistochemical staining for Figure 5/Immunohistochemical staining/SMA/Mod/Mod-1-400X.jpg]

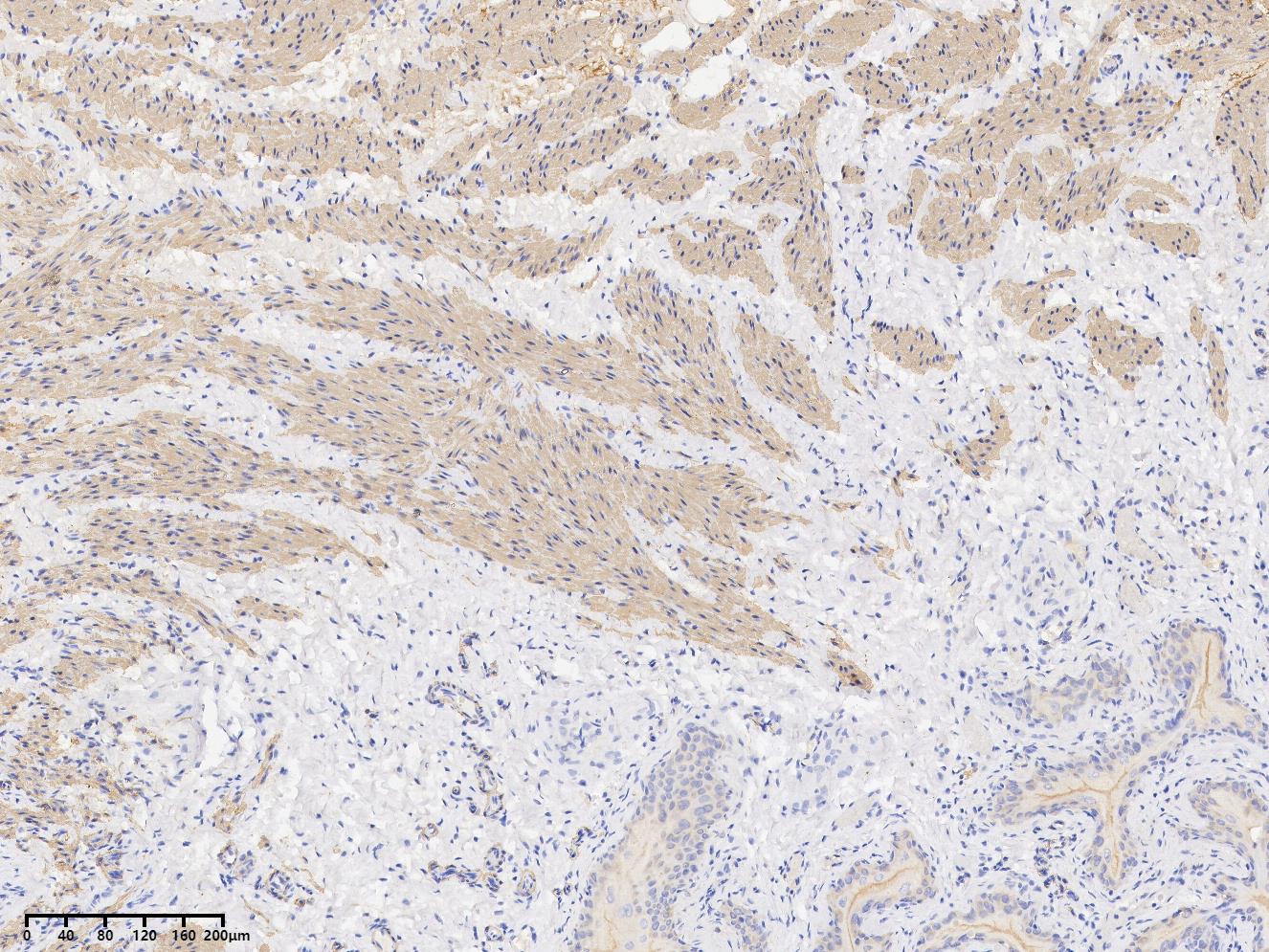

Supplement: Supplemental Information 4 [file peerj-13-20186-s004.zip › Raw date-HE and Immunohistochemical staining for Figure 5/Immunohistochemical staining/SMMHC/Con-1/Con-1-100X.jpg]

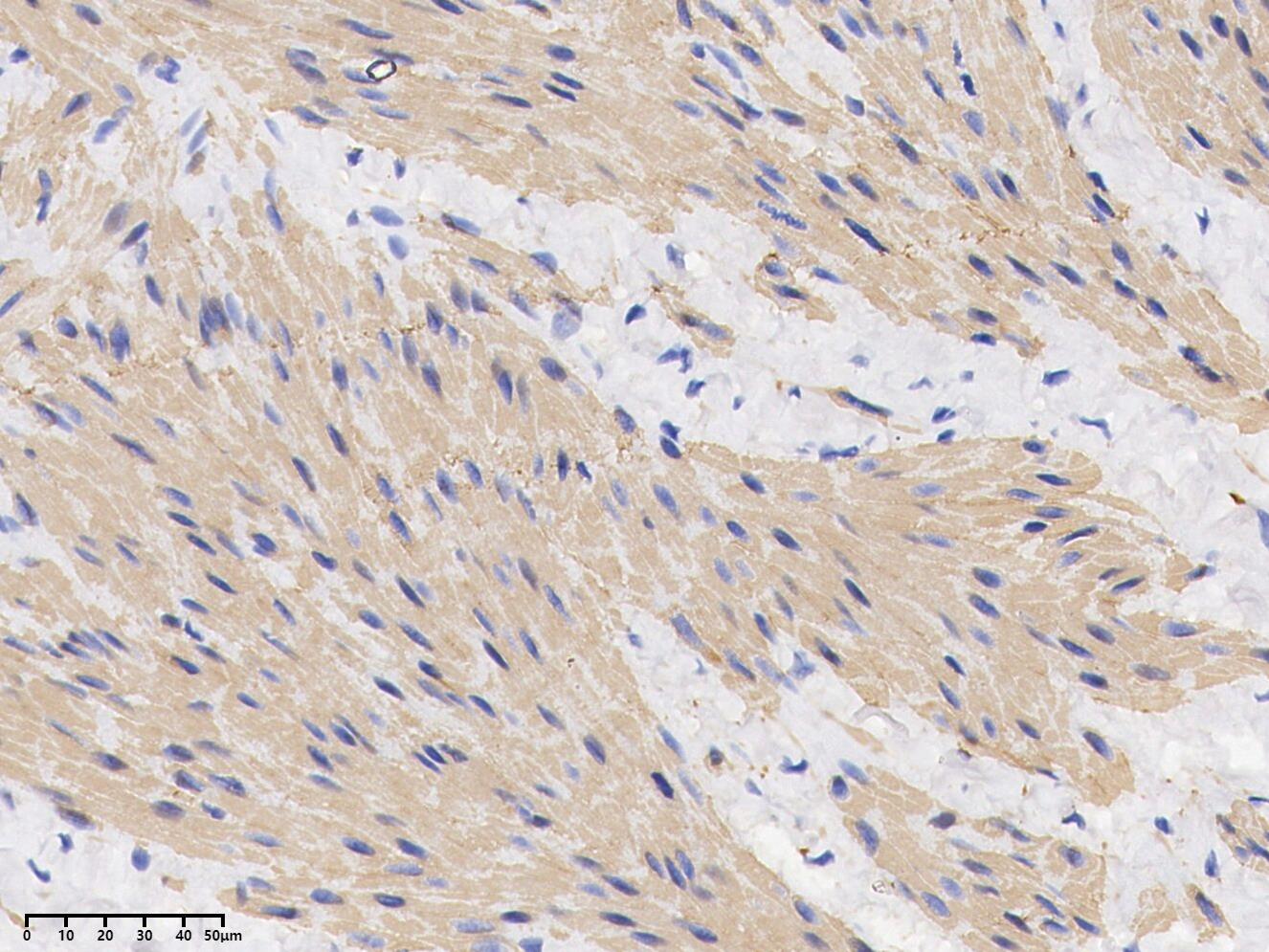

Supplement: Supplemental Information 4 [file peerj-13-20186-s004.zip › Raw date-HE and Immunohistochemical staining for Figure 5/Immunohistochemical staining/SMMHC/Con-1/Con-1-400X.jpg]

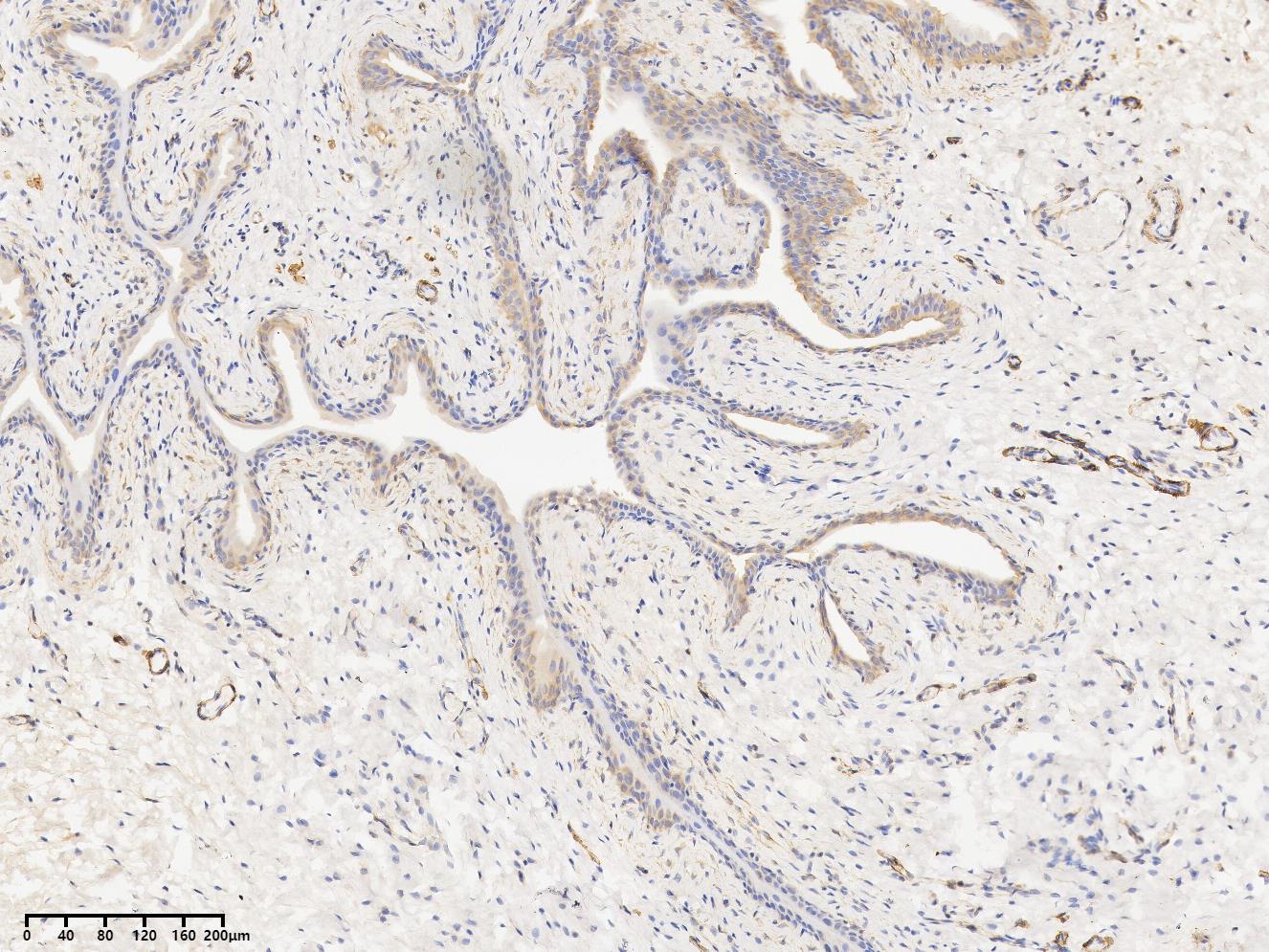

Supplement: Supplemental Information 4 [file peerj-13-20186-s004.zip › Raw date-HE and Immunohistochemical staining for Figure 5/Immunohistochemical staining/SMMHC/Mod/Mod-1-100X.jpg]

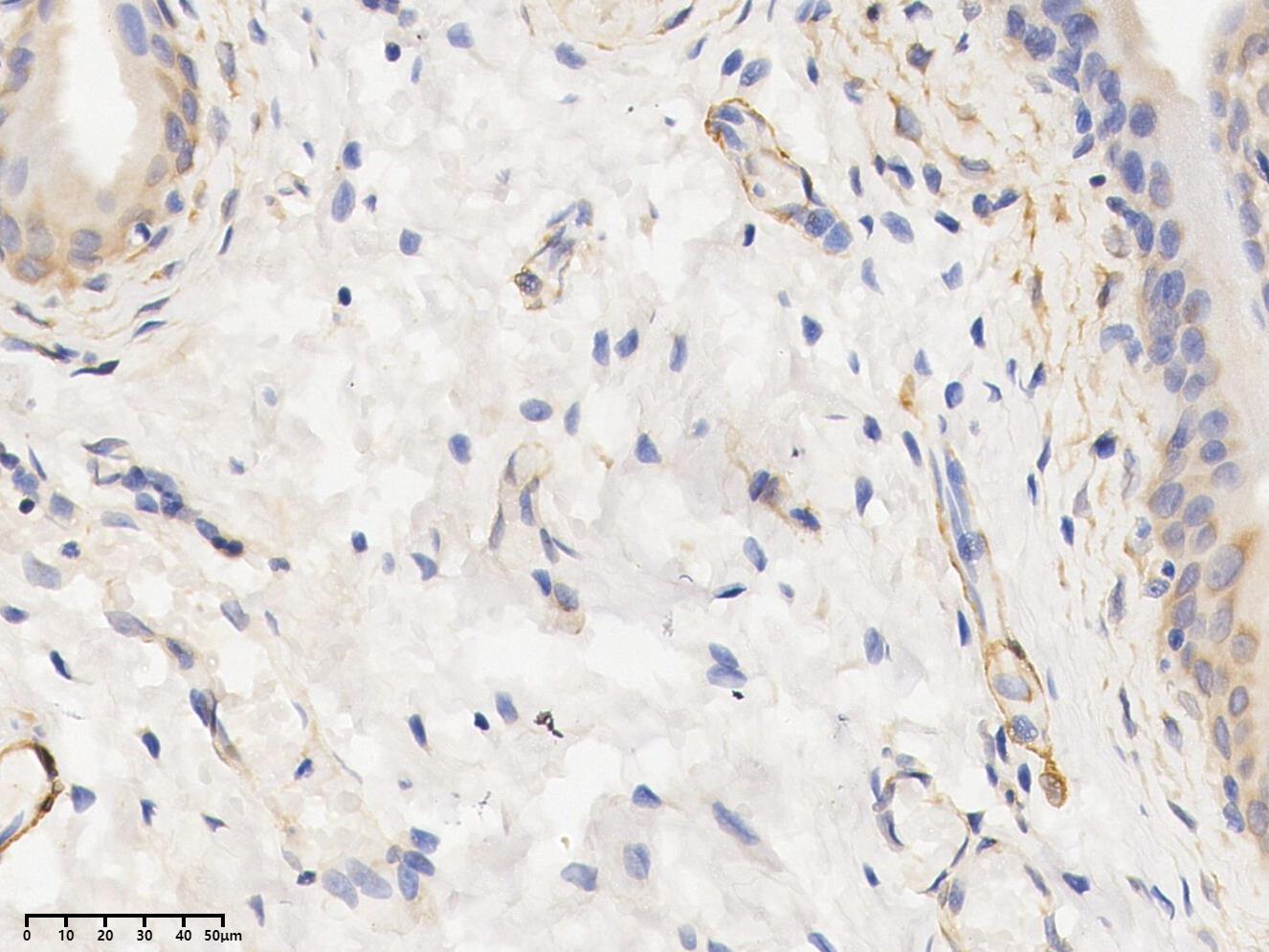

Supplement: Supplemental Information 4 [file peerj-13-20186-s004.zip › Raw date-HE and Immunohistochemical staining for Figure 5/Immunohistochemical staining/SMMHC/Mod/Mod-1-400X.jpg]

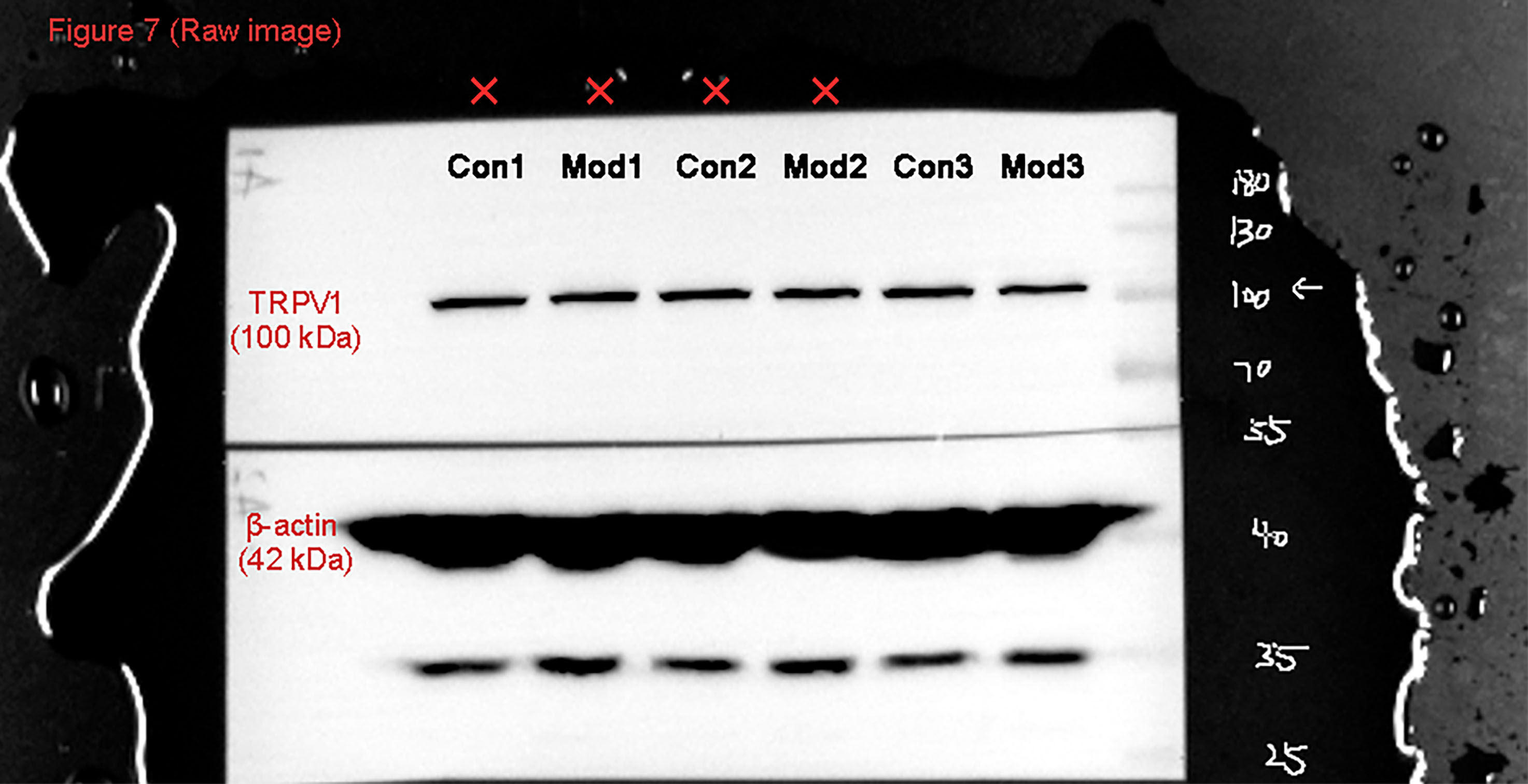

Supplement: Supplemental Information 6 [file peerj-13-20186-s006.jpg]

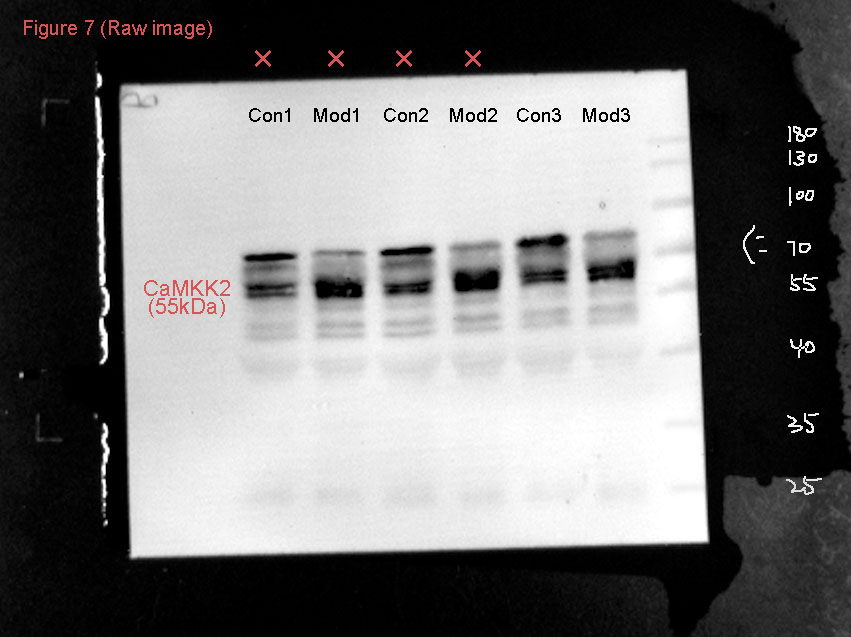

Supplement: Supplemental Information 7 [file peerj-13-20186-s007.jpg]

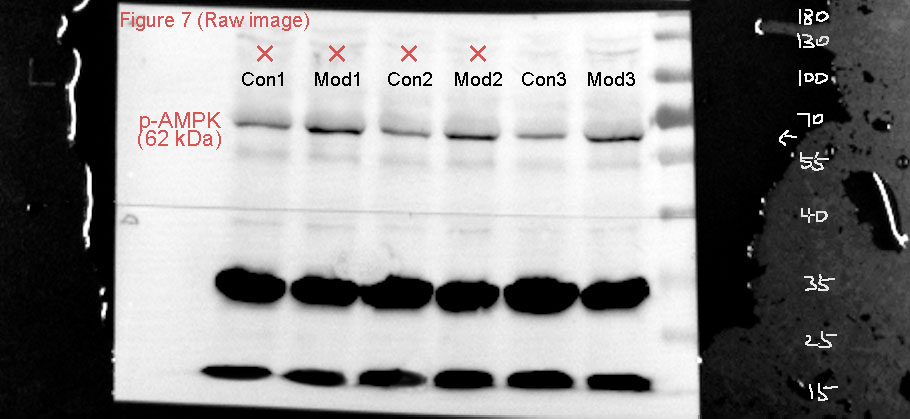

Supplement: Supplemental Information 8 [file peerj-13-20186-s008.jpg]

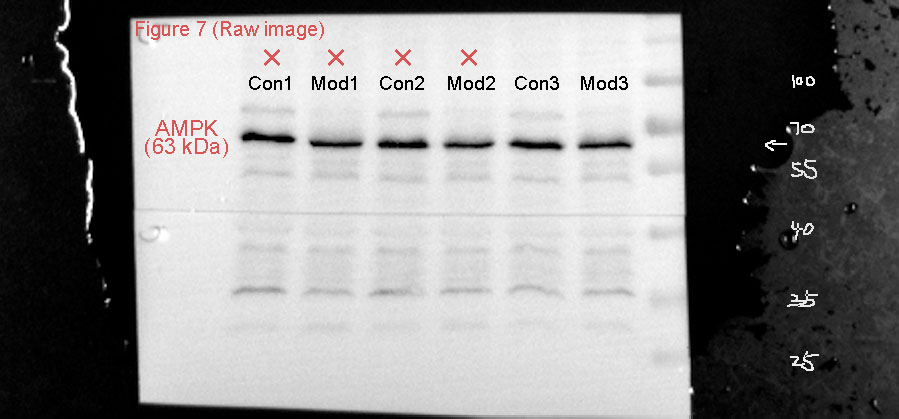

Supplement: Supplemental Information 9 [file peerj-13-20186-s009.jpg]

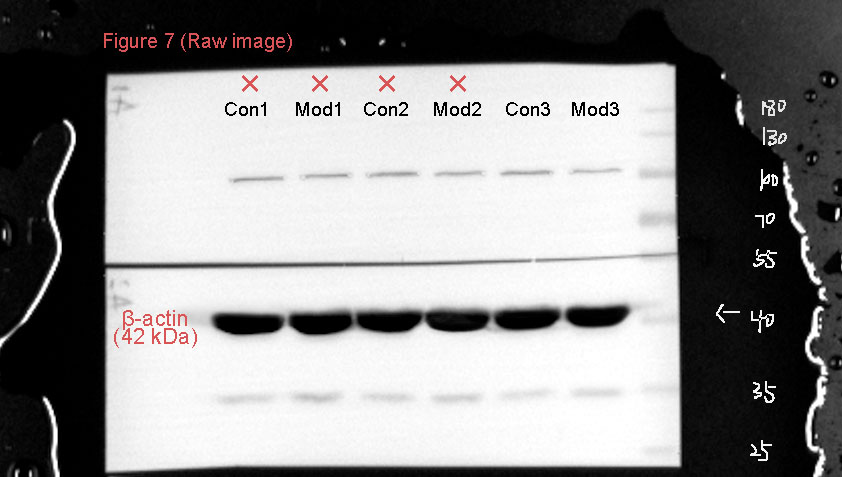

Supplement: Supplemental Information 10 [file peerj-13-20186-s010.jpg]
